# Quantifying the source–sink balance and carbohydrate content in three tomato cultivars

**DOI:** 10.3389/fpls.2015.00416

**Published:** 2015-06-05

**Authors:** Tao Li, Ep Heuvelink, Leo F. M. Marcelis

**Affiliations:** ^1^Horticulture and Product Physiology Group, Department of Plant Sciences, Wageningen University, WageningenNetherlands; ^2^Institute of Environment and Sustainable Development in Agriculture, Chinese Academy of Agriculture Science, BeijingChina

**Keywords:** source–sink balance, plant development stage, carbohydrate content, quantification, tomato cultivars, *Solanum lycopersicum*

## Abstract

Supplementary lighting is frequently applied in the winter season for crop production in greenhouses. The effect of supplementary lighting on plant growth depends on the balance between assimilate production in source leaves and the overall capacity of the plants to use assimilates. This study aims at quantifying the source–sink balance and carbohydrate content of three tomato cultivars differing in fruit size, and to investigate to what extent the source/sink ratio correlates with the potential fruit size. Cultivars Komeet (large size), Capricia (medium size), and Sunstream (small size, cherry tomato) were grown from 16 August to 21 November, at similar crop management as in commercial practice. Supplementary lighting (High Pressure Sodium lamps, photosynthetic active radiation at 1 m below lamps was 162 μmol photons m^-2^ s^-1^; maximum 10 h per day depending on solar irradiance level) was applied from 19 September onward. Source strength was estimated from total plant growth rate using periodic destructive plant harvests in combination with the crop growth model TOMSIM. Sink strength was estimated from potential fruit growth rate which was determined from non-destructively measuring the fruit growth rate at non-limiting assimilate supply, growing only one fruit on each truss. Carbohydrate content in leaves and stems were periodically determined. During the early growth stage, ‘Komeet’ and ‘Capricia’ showed sink limitation and ‘Sunstream’ was close to sink limitation. During this stage reproductive organs had hardly formed or were still small and natural irradiance was high (early September) compared to winter months. Subsequently, during the fully fruiting stage all three cultivars were strongly source-limited as indicated by the low source/sink ratio (average source/sink ratio from 50 days after planting onward was 0.17, 0.22, and 0.33 for ‘Komeet,’ ‘Capricia,’ and ‘Sunstream,’ respectively). This was further confirmed by the fact that pruning half of the fruits hardly influenced net leaf photosynthesis rates. Carbohydrate content in leaves and stems increased linearly with the source/sink ratio. We conclude that during the early growth stage under high irradiance, tomato plants are sink-limited and that the level of sink limitation differs between cultivars but it is not correlated with their potential fruit size. During the fully fruiting stage tomato plants are source-limited and the extent of source limitation of a cultivar is positively correlated with its potential fruit size.

## Introduction

Plant growth is closely correlated with source and sink strength and the balance between them (Gifford and Evans, 1981; Wardlaw, 1990; Smith and Stitt, 2007). Source strength of a plant is defined as the rate at which the plant produces assimilates (photosynthesis rate). The sink strength of a plant is composed of sink strengths of all individual organs. Sink strength of an organ is the competitive ability of an organ to attract assimilates and can be quantified by its potential growth rate (Marcelis, 1996). Although fruits are the major sink organs in crops like tomato, also leaves, stems, and roots utilize assimilates and have a sink strength; hence leaves are not only source organ but also sink organ.

Source–sink balance regulates carbon status in plants (Osorio et al., 2014). Differences in source–sink balance are expected to result in differences in carbohydrate content in plants (Paul and Foyer, 2001; Dingkuhn et al., 2007; Patrick and Colyvas, 2014). In a source-limited situation, carbohydrate content in the plants might be low as plants have sufficient sinks to utilize the produced assimilates. However, in a sink-limited situation plant growth cannot keep pace with assimilate production. When assimilate production exceeds its utilization carbohydrates (starch and soluble sugars) are usually stored in leaves (Yelle et al., 1989) as well as stems (Hocking and Steer, 1994; Scofield et al., 2009). Limited sink demand could result in feedback regulation of photosynthesis as it may down-regulate the net photosynthetic activity through carbohydrate accumulation in source leaves (Iglesias et al., 2002; Franck et al., 2006; McCormick et al., 2006; Velez-Ramirez et al., 2014).

Manipulating source and sink organs (e.g., fruit and leaf pruning) are often applied to investigate plant source–sink balance (Cockshull and Ho, 1995; Iglesias et al., 2002; Matsuda et al., 2011). Crop growth models can be used to quantify the source and sink strength (De Koning, 1994; Heuvelink, 1996b; Wubs et al., 2009, 2012). In these models the sink strength of a growing organ is determined by its potential growth rate (i.e., growth under non-limiting assimilate supply; Marcelis, 1996), which depends on its developmental stage (Marcelis and Baan Hofman-Eijer, 1995). Cumulating the sink strength of each organ on the plant results in total plant sink strength. The plant source strength is calculated as the supply of assimilates during a day, which is estimated by the crop growth rate (g dry mass plant^-1^ day^-1^; Heuvelink, 1995).

The growth environment plays a pivotal role in determining the source–sink balance. Under non-stressing conditions, irradiance becomes particularly important as it is the driving force for photosynthesis. Supplementary lighting is commonly applied in greenhouses in order to improve crop photosynthesis and thus production (Heuvelink et al., 2006; Moe et al., 2006). The beneficial effect of supplementary lighting is determined by the balance between assimilate production in source leaves and the overall capacity of the plants to use these assimilates. This implies that it is important to identify the plant source–sink balance in order to efficiently utilize supplementary lighting.

The source–sink balance of a plant varies significantly during its life span because of the continuous organ initiation and development which affects both the sink and source strength (Wardlaw, 1990). During the early growth stage, tomato plants might be prone to sink limitation as there might be insufficient sinks to utilize all the produced assimilates. This might occur especially under high irradiance. During the reproductive stage, tomato plants generally bear many fruits, and assimilate supply might not meet the sink demand. This has been suggested in studies where fruit pruning increased fruit size of the remaining fruits without influencing the total plant biomass production (Cockshull and Ho, 1995; Heuvelink, 1996b; Matsuda et al., 2011). Tomato source–sink balance could also differ between cultivars which often differ in fruit load and potential fruit growth rate, suggesting differences in sink strength (Heuvelink and Marcelis, 1989; Marcelis, 1996). Cultivars may also differ in source strength as leaf photosynthetic properties, leaf area and plant architecture may differ. Dueck et al. (2010) observed that under commercial crop management effects of supplementary lighting were small in cherry tomato compared with cultivars with large-sized fruits. They argued that cherry tomato had less sink demand although it bears more fruits. A detailed analysis of the source–sink balance from early growth stage to fully fruiting stage for cultivars with different potential fruit size has not performed so far.

The objectives of this study are to provide a detailed quantitative analysis of source–sink balance as well as carbohydrate content of tomato plants with standard fruit load during their development; and to investigate to what extent the source/sink ratio of a cultivar depends on the potential fruit size. Our hypotheses are (1) tomato plants are sink-limited during their early growth stage when grown under high irradiance; (2) tomato plants are source-limited during the fully fruiting stage, and the source/sink ratio negatively correlates with the potential fruit size (when comparing cultivars at their commercial fruit load). To test these hypotheses, three types of tomato cultivars with different potential fruit size were grown under conditions comparable to commercial crop management from mid-August until end of November. The source/sink ratio and carbohydrate content were examined during this period through experimental observation combined with model estimation.

## Materials and Methods

### Plant Materials and Growth Conditions

Tomato (*Solanum lycopersicum*) plants were planted in a Venlo-type glasshouse compartment on 16 August and grown until 21 November 2013. The greenhouse compartment had an area of 150 m^2^ with a gutter height of 5 m, and was located in Wageningen, the Netherlands (52°N, 5°E). Eight growth gutters were evenly arranged in the compartment in the East to West direction with a distance of 150 cm between gutters. Plants on each gutter were alternatively trained to two high wires which were 30 cm to the right and left of the growth gutter. Forty-five plants were grown on each gutter at an inter-plant distance of 20 cm. All plants were grown with single shoot. Plant density was initially 3.3 plants m^-2^ and gradually decreased to 2.2 plants m^-2^ at the end of the experiment due to periodical destructive harvests. Plants were grown on Rockwool with drip irrigation according to the commercial practice. From 43 days after planting onward, leaves below the second lowest truss were regularly removed. Fruits were picked when they turned red-ripe.

**FIGURE 1:**
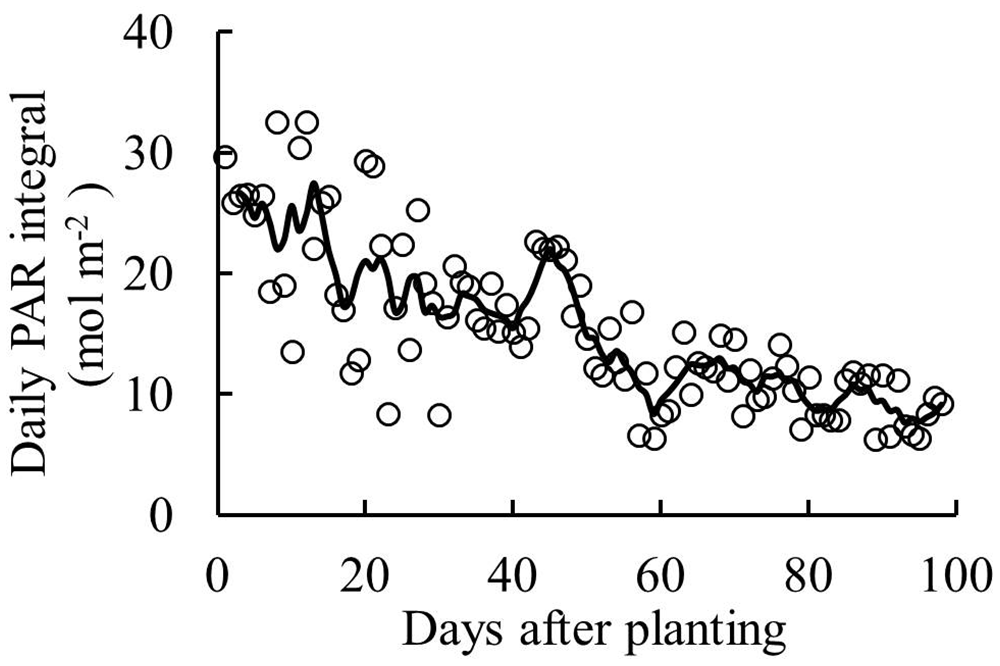
**Daily PAR integral inside the greenhouse (sum of natural irradiance and supplementary lighting) during the experiment**. Line represents moving average over 5 days.

Solar radiation was continuously measured outside the greenhouse throughout the experimental period. PAR was estimated from solar radiation, assuming half the global radiation is PAR (Jacovides et al., 2003). Greenhouse transmissivity of PAR was 62%. Supplementary lighting (High Pressure Sodium lamps, HortiluxSchreder, HPS600W/400V) was applied from 19 September until the end of the experiment. PAR of the supplementary lighting was 162 ± 9 μmol photons m^-2^ s^-1^ at 1 m below the lamps. The lamps were turned on when global radiation was below 200 W m^-2^ and turned off when it exceeded 300 W m^-2^ between 6:00 to 16:00 h. A standard greenhouse computer (Hoogendoorn-Economic, Hoogendoorn, Vlaardingen, the Netherlands) was used to control the greenhouse climate as well as supplementary lighting. Sunrise to sunset at start of the experiment was from 6:30 to 21:00, it was from 8:00 to 16:40 at end of the experiment. During the experiment, average daily outside global radiation was 9 MJ m^-2^ d^-1^; inside the greenhouse, average day/night temperature was 24/18°C, air humidity was 77% and day time CO_2_ concentration was 577 μmol mol^-1^. Daily PAR integral inside the greenhouse is presented in **Figure 1**.

### Treatments

Three tomato cultivars with different potential fruit size and with standard fruit load were grown on eight gutters (double rows) in the same greenhouse in order to compare their source–sink balance during plant development: cv. Komeet (large size, five fruits per truss), Capricia (medium size, six fruits per truss), and Sunstream (small size, 10 fruits per truss). Additionally, a set of plants of these cultivars were pruned to one fruit per truss, in order to determine the potential growth rate of a single fruit which is an estimate of sink strength of a single fruit (Marcelis, 1996). Furthermore, another set of plants of all cultivars were pruned to half fruit load: cv. Komeet (two fruits per truss), Capricia (three fruits per truss), Sunstream (five fruits per truss), in order to determine the effect of reduced sink strength on total biomass and net leaf photosynthesis.

The greenhouse was divided into three equal parts, perpendicular on the gutters: at the West side the tallest cultivar (Sunstream) was grown, at the East side the smallest cultivar (Capricia) was grown, and in the middle cultivar Komeet was grown. For each of the six central gutters, six plants were grown with standard fruit load and one with half fruit load for each cultivar. The number of plants with standard fruit load was larger than those at half fruit load as for standard fruit load destructive measurements were taken at six moments while for half fruit load these measurements were only performed at the end of the experiment. Each plant with standard and half fruit load was surrounded on both sides by an internal border plant. All plants on the two outer gutters as well as the internal border plants were pruned to one fruit per truss. Fruit pruning was done immediately after fruit set for each truss.

### Plant Development Registration

Observations on flowering and fruit age were taken three times a week. Flowering was defined as three fully open flowers on a truss, which indicates fruit age 0. For the treatment with standard fruit load, 12 plants of each cultivar which were used for the last two destructive harvests were investigated. This observation was used for estimating the sink strength of the plant with standard fruit load. Due to more plants were available for the treatment with one fruit per truss, observations on flowering and fruit age of this treatment were taken on 15–20 plants of each cultivar. Furthermore, the maximum fruit length and diameter of the fruits from the treatment with one fruit per truss were measured with caliper three times a week since fruit set in order to obtain fruit volume over time, number of measured fruits ranged from 34 to 48 fruits per cultivar, these fruits were from the first three trusses which developed in September. The observation of fruit volume and fruit age of the treatment with one fruit per truss was used for estimating the potential growth rate of a single fruit. Total formed truss number was 11, 11, and 14 for Komeet, Capricia, and Sunstream, respectively, until the end of the experiment. Plant development registration was not performed in the treatment with half fruit load due to sink strength of this treatment was not addressed.

Fruit set started between 20–30 days after planting for the three cultivars. Therefore, the first 30 days after planting was defined as early growth stage, since 30 days after planting onward was defined as fully fruiting stage.

### Destructive Measurements

Six plants per cultivar were destructively measured before planting (on 15 August) to determine their initial total biomass and leaf area. For the plants with standard fruit load six plants of each cultivar (one from each gutter) were harvested on 18, 33, 47, 61, 81, 97 days after planting. For plants with half fruit load six plants (one from each gutter) were harvested on 97 days after planting. Fresh and dry weight of leaves, stems, and fruit trusses were determined. Plant organs were dried for at least 48 h at 105°C in a ventilated oven. Leaf area was measured with a leaf area meter (LI-3100C, LI-COR Inc., Lincoln, NE, USA). SLA was calculated by dividing leaf area by leaf dry weight. The regularly removed leaves and harvested fruits were dried and dry weight was added to obtain the cumulative dry weights per plant; area of the regularly removed leaves was also determined for estimating total LAI at different moments which was needed as model input.

For each cultivar, 97–148 fruits from the plants with one fruit per truss were randomly sampled during the experiment, the samples were taken once per week, and fruit diameter, length, age, fresh, and dry weight were recorded. These observations were used to get two relationships: a relationship between fruit volume and fresh weight; and a relationship between fruit age and fruit dry matter content.

### Sample Collection and Carbohydrates Analysis

Leaf and stem samples for carbohydrate analyses were taken from plants with standard fruit load. Leaf samples were taken at the beginning of the day (6:00–7:00 AM) at 1 day before each destructive harvest. The samples were taken at every other leaf from leaf number 5 (uppermost fully expanded leaf; leaf number 1 was the uppermost leaf longer than 5 cm) downward to the bottom of the canopy. In each selected leaf, one leaflet adjacent to the terminal leaflet was collected. The collected leaflets from one plant were pooled together to represent one canopy leaf sample. Stem samples were taken on the day of destructive harvest. Stem sections (0.5 cm length) were taken from top to the bottom where the leaf samples were taken, these sections were pooled together to represent one stem sample. Six replicates were taken for each type of sample at each time. Fresh weight of all collected samples was determined and added to the total plant weight.

Samples were inserted in vials and flash-frozen in liquid nitrogen. They were transferred to a freezer (−80°C) for storage. Starch and soluble sugar content were analyzed with a HPLC Dionex system (GS 50 pump and PED 2 electrochemical detector) as described by Savvides et al. (2014); the soluble sugars that were monitored were fructose, glucose, and sucrose.

### Net Photosynthesis Measurements

Net photosynthesis rates were measured with a portable gas exchange device equipped with a leaf chamber fluorometer (LI-6400; LI-COR) at leaf number 6 from top of the canopy. In the measurement chamber, PAR (10% blue, 90% red) was 1000 μmol m^-2^ s^-1^, CO_2_ concentration was 500 μmol mol^-1^, air temperature was 23°C, and vapor pressure deficit (VPD) was between 0.5–1 kPa. The measurements were performed on plants with standard fruit load as well as plants with half fruit load on 20, 28, 39, 54–55, 64–65, and 75–76 days after planting (plants with half fruit load only from 54 days onward). For each cultivar each time six measurements were taken before noon (between 8:30 and12:00) and six were taken after noon (between 12:30 and 16:00).

### Plant Source/Sink Ratio Determination

Source/sink ratio was estimated based on source strength of the plant divided by the sum of the vegetative sink strength and total fruit sink strength.

Plant growth rate (g dry mass plant^-1^ day^-1^) was used as an estimate of source strength. Daily plant growth rate was estimated by the crop growth model TOMSIM (Heuvelink, 1996b) with measured SLA (from planting date to first destructive harvest date), measured LAI (from first destructive harvest date onward), dry matter partitioning among plant organs (leaves, fruits, stems, roots), and the climate data (global radiation, intensity and timing of the supplementary lighting, greenhouse temperature, and CO_2_) were input to the model. The fraction dry matter partitioned to roots was set to 13% at planting; and 4% from first fruit harvest onward; in between this fraction was estimated by linear interpolation (Heuvelink, 1995). Estimated daily plant growth rate was multiplied by a correction factor such that estimated cumulative plant weights corresponded to the measured cumulative plant weights. This factor was estimated by minimizing the sum of squares of the residuals between measured and estimated total dry weight at each destructive harvest (one factor for each cultivar).

Sink strength of a single fruit, quantified by the potential fruit growth rates, was obtained by non-destructive measurements on potentially growing fruits (i.e., one fruit per truss). On the basis of the lengths and diameters of the potentially growing fruits, their volume was calculated assuming a deformed sphere

(1) $$\nu =\frac{4}{3}\text{ π}{\left(\frac{d}{2}\right)}^{2}\frac{h}{2}\;$$

where, *v* is fruit volume (cm^3^), *d* is fruit diameter (cm), *h* is fruit length (cm).

Fruit volume was subsequently converted into fresh weight, using a cultivar specific linear regression between fruit volume and fruit fresh weight (*r*^2^ = 0.97–0.99 for three cultivars). A Gompertz function was fitted through fresh weight over time

(2) $$w(t)=w_{max}e^{-e^{-k(t-t_{m})}}\;$$

where, *w*(*t*) is the weight at age *t* (*d* after anthesis), *w_max_* is upper asymptote of fruit weight (g), *k* represents the weighted mean relative growth rate, and *t_m_* the age (d) at maximum growth rate.

The Gompertz function was fitted through the data with non-linear mixed modeling. Non-linear mixed models take into account that the measurements on one fruit are grouped. A lower variation is assumed between the measurements of one fruit than between the measurements of different fruits. The three parameter means (*w_max_*, *t_m_*, *k*) were estimated to describe fruit growth (Wubs et al., 2009).

A fourth-order polynomial function was fitted for the destructively determined fruit dry matter content as a function of fruit age according to Wubs et al. (2012). The potential growth rate in g dry matter per day of an individual fruit (representing the sink strength of a single fruit) was calculated as the product of the derivative of the Gompertz function for fruit fresh weight and this fourth-order polynomial function. The daily total fruit sink strength of a plant was calculated by accumulating the sink strength of all fruits which were present on the plant that day.

Vegetative sink strength was estimated as the integral of sink strengths of each vegetative unit (De Koning, 1994; Heuvelink, 1996b).

(3) $$PVGR=ae^{-0.168(T-19)}PFGR\;$$

where, *PVGR* is the potential growth rate for a vegetative unit (g d^-1^) and *PFGR* is the potential fruit growth rate (g d^-1^) for a single fruit. *a* is a specific factor between potential fruit growth rate and potential growth rate of a vegetative unit, which was estimated by minimizing the sum of squares of the residuals between measured and estimated dry matter partitioning to fruits, the latter was calculated as estimated fruit dry matter divided by cumulative plant dry matter; this factor is cultivar dependent. *T* is the average greenhouse diel temperature during the experiment period (°C).

Before anthesis of the first truss, vegetative growth is an input. Usually about three vegetative units precede the first truss (Dieleman and Heuvelink, 1992), which was also observed in this experiment. The sink strengths of these three units were estimated by using *PVGR* multiplied by three specific factors [0.6, 0.75, 0.9, respectively, from the first to the third unit, these factors were derived based on Heuvelink (1996a)], this is because the first few units are relatively small and hence have a low sink strength. The daily total vegetative sink strength of a plant was calculated by accumulating the vegetative sink strength of all units which were present that day. A more detailed description see De Koning (1994) and Heuvelink (1996a).

### Statistical Analysis

Destructive measurements and carbohydrate determination were based on six replicate plants; net leaf photosynthesis was based on 12 replicates (two leaves per plant, six replicate plants). The effects of cultivars, days after planting, and fruit pruning treatments on measured plant parameters were evaluated by ANOVA followed by Fisher’s protected least significant difference test (l.s.d) at 95% confidence, using GenStat 16th edition.

## Results

### Plant Growth

**FIGURE 2:**
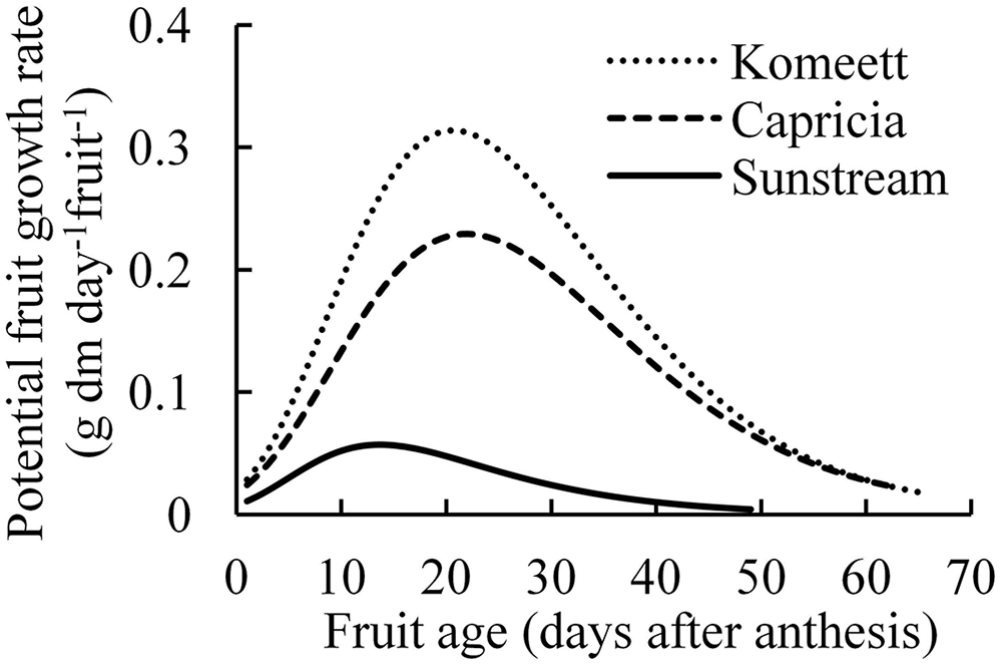
**Potential growth rate of individual fruits for three tomato cultivars**. Curves end at the average growth duration (time from anthesis until harvest ripe) of each cultivar. Number of measured fruits ranged from 34 to 48 fruits per cultivar. Potential growth was created by maintaining only one fruit per truss.

Maximum growth rate and growth duration of single fruit were highest in ‘Komeet’; while these parameters were lowest in ‘Sunstream’ (**Figure 2**). These differences together resulted in the largest potential fruit size in ‘Komeet’ and smallest in ‘Sunstream.’ Potential fresh fruit weight was 180 *g* for ‘Komeet,’ 137 *g* for ‘Capricia,’ and 20 *g* for ‘Sunstream’ as determined in this study.

**FIGURE 3:**
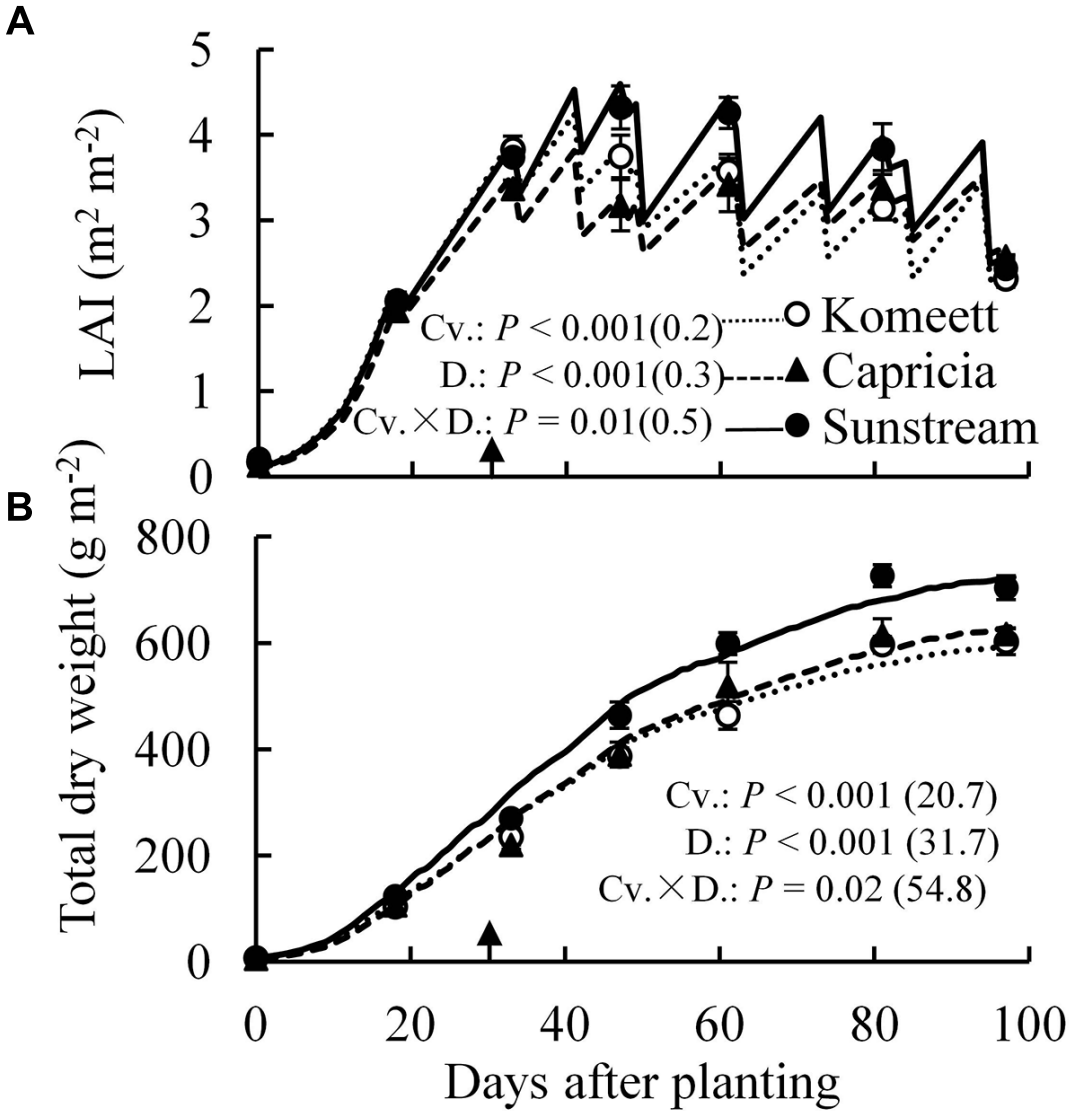
**Measured (symbols) and estimated (lines) LAI **(A)** and total dry weight **(B)** over time for three tomato cultivars with standard fruit load**. Error bars through data points show ±SE (*n* = 6). The result of two-way ANOVA with cultivar (Cv.) and days after planting (D.) as independent variables and their interaction (Cv. × D.) for each dependent variable is shown in each panel. The value in the bracket indicates the least significant difference at *P* = 0.05 (l.s.d). Arrow in X-axis indicates 30 days after planting. Fruit set started between 20–30 days after planting for the three cultivars. Therefore, the left side of arrow was defined as early growth stage, the right side of arrow was defined as fully fruiting stage.

‘Sunstream’ had highest LAI during a large part of the growing period (**Figure 3A**), and highest total dry weight except for the initial period after planting (**Figure 3B**); while these parameters were similar between ‘Komeet’ and ‘Capricia’ (**Figure 3**). For all cultivars, plant total dry weight was not affected by the half fruit load treatments (**Table 1**). However, half fruit load treatments resulted in significantly higher fraction of dry mass partitioned to leaves and stems, and lower partitioning to fruits (**Table 1**).

**Table 1:** Plant total dry mass and fraction of dry mass partitioned to leaves, stems and fruits of three tomato cultivars in response to fruit pruning treatment (data are collected at the end of the experiment, *n* = 6).

Treatment	Total dry weight (g plant^-1^)	Dry mass partitioning (%)		
		Leaves	Stems	Fruits
**‘Komeet’**
Standard fruit load	271.5 (±11)^a^	37.9 (±1.4)^a^	16.3 (±0.4)^a^	45.8 (±1.6)^b^
Half fruit load	275.1 (±10)^a^	42.3 (±0.7)^b^	20.2 (±0.5)^b^	37.5 (±1.0)^a^
**‘Capricia’**
Standard fruit load	278.2 (±5)^a^	36.3 (±1.0)^a^	17.3 (±0.6)^a^	46.4 (±1.4)^b^
Half fruit load	277.0 (±16)^a^	41.0 (±0.9)^b^	19.5 (±0.5)^b^	39.5 (±0.7)^a^
**‘Sunstream’**
Standard fruit load	317.3 (±10)^b^	45.2 (±0.5)^a^	20.1 (±0.4)^a^	34.7 (±0.8)^b^
Half fruit load	316.4 (±17)^b^	52.7 (±0.3)^b^	25.1 (±0.5)^b^	22.2 (±0.6)^a^

*Means followed by different letters within one column of each cultivar differ significantly as established by the least significant difference (l.s.d) test at *P* = 0.05.*

### Carbohydrate Content and Net Photosynthesis Rate

**FIGURE 4:**
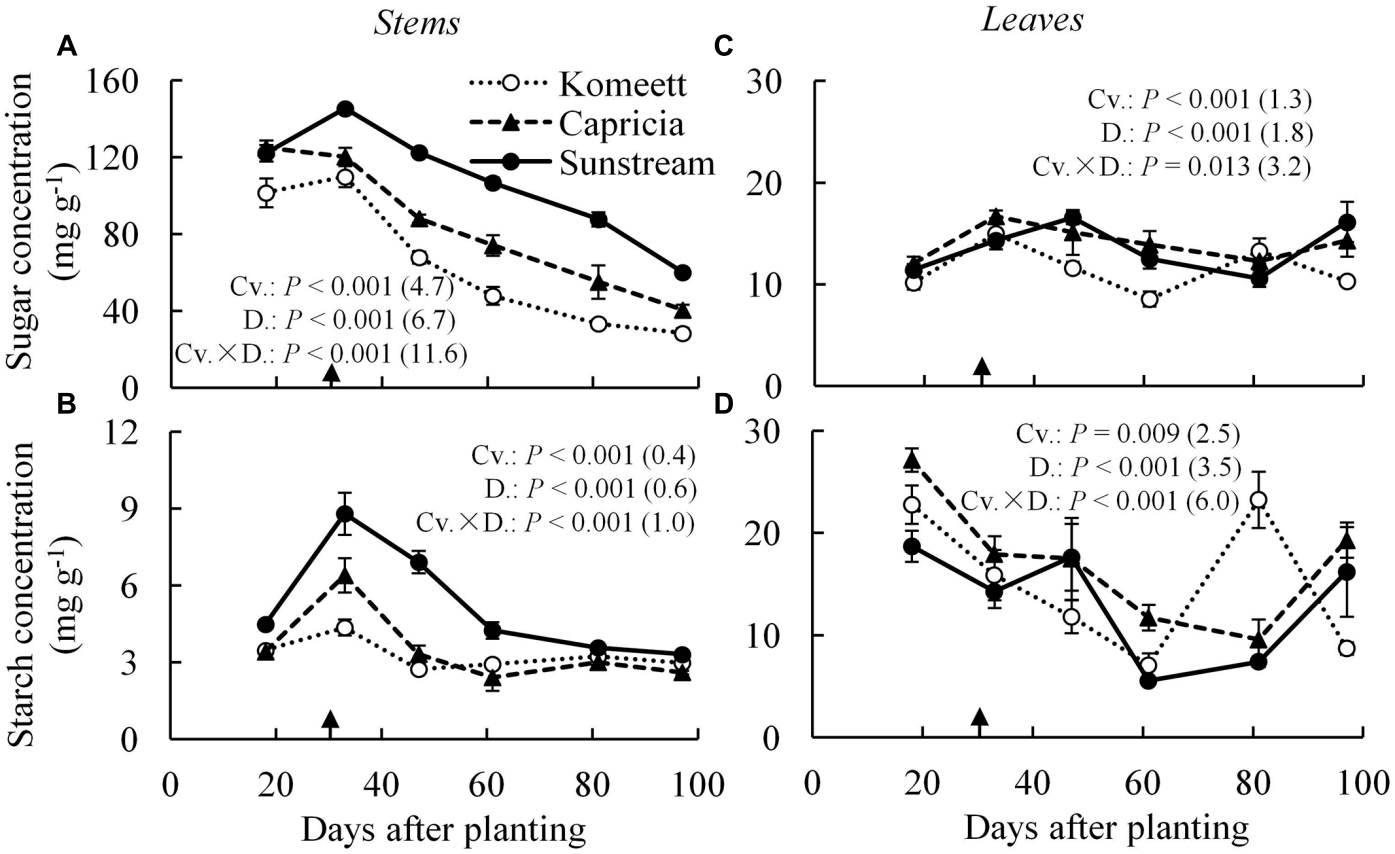
**Time course of the soluble sugar **(A,C)** and starch **(B,D)** concentration in the stems **(A,B)** and leaves **(C,D)** of three tomato cultivars with standard fruit load**. Soluble sugar is the sum of glucose, fructose, and sucrose. Error bars through data points show ±SE (*n* = 6). The result of two-way ANOVA with cultivar (Cv.) and days after planting (D.) as independent variables and their interaction (Cv. × D.) for each dependent variable is shown in each panel. The value in the bracket indicates the least significant difference at *P* = 0.05 (l.s.d). Arrow in X-axis indicates 30 days after planting. Fruit set started between 20–30 days after planting for the three cultivars. Therefore, the left side of arrow was defined as early growth stage, the right side of arrow was defined as fully fruiting stage.

In tomato stems, starch content was negligible compared to sugar content which was apparently the main carbohydrate in stems (**Figures 4A,B**). For all cultivars, soluble sugar content was at a high level until 33 days after planting. Thereafter, it decreased gradually until the end of the experiment (**Figure 4A**). This phenomenon was not observed for starch content which reached a peak at 33 days after planting for ‘Capricia’ and ‘Sunstream,’ and remained relatively constant from 60 days after planting onward for all three cultivars (**Figure 4B**). ‘Sunstream’ had higher sugar content than the other two cultivars (*P* < 0.001) except for at 18 days after planting (**Figure 4A**); it also had highest starch content (*P* < 0.001; **Figure 4B**).

In leaves, soluble sugar content was relatively constant during the growing period compared to starch content (**Figures 4C,D**). For all cultivars, starch content was initially (18 days after planting) high and decreased gradually until 60 days after planting. Surprisingly, starch content at 80 days after planting suddenly increased and reached a level as high as that observed at 18 days after planting in ‘Komeet.’ At the end of the experiment, starch content increased in ‘Capricia’ and ‘Sunstream’ (**Figure 4D**).

**FIGURE 5:**
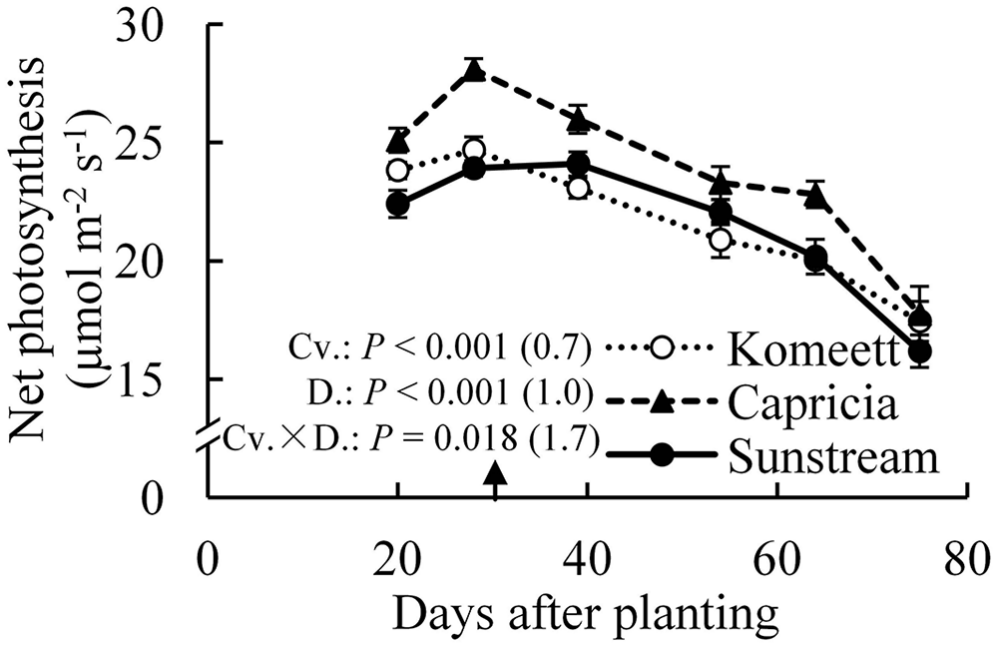
**Time course of the net photosynthesis rate of leaf number six from top of the canopy in the three tomato cultivars with standard fruit load**. In the measurement chamber, light intensity, CO_2_ concentration, air temperature, and VPD were maintained at 1000 μmol m^-2^ s^-1^, 500 μmol mol^-1^, 23°C, and between 0.5–1 kPa. Error bars through data points show ±SE (*n* = 12). The result of two-way ANOVA with cultivar (Cv.) and days after planting (D.) as independent variables and their interaction (Cv. × D.) for each dependent variable is shown in the figure. The value in the bracket indicates the least significant difference at *P* = 0.05 (l.s.d). Arrow in X-axis indicates 30 days after planting. Fruit set started between 20–30 days after planting for the three cultivars. Therefore, the left side of arrow was defined as early growth stage, the right side of arrow was defined as fully fruiting stage.

For all cultivars, the highest net photosynthesis rates were observed at 28 days after planting; thereafter it decreased gradually until the end of the experiment (**Figure 5**). Interestingly, net photosynthesis rates at 20 days after planting were tended to be lower than at 28 days after planting, although this difference was only significant in ‘Capricia’ (**Figure 5**). Furthermore, ‘Capricia’ had higher net photosynthesis rates than the other two cultivars (*P* < 0.001). Half fruit pruning treatments had no effect on net photosynthesis rates in all three cultivars (data not shown).

### Source–Sink Balance and its Relationship with Plant Carbohydrate Content

**FIGURE 6:**
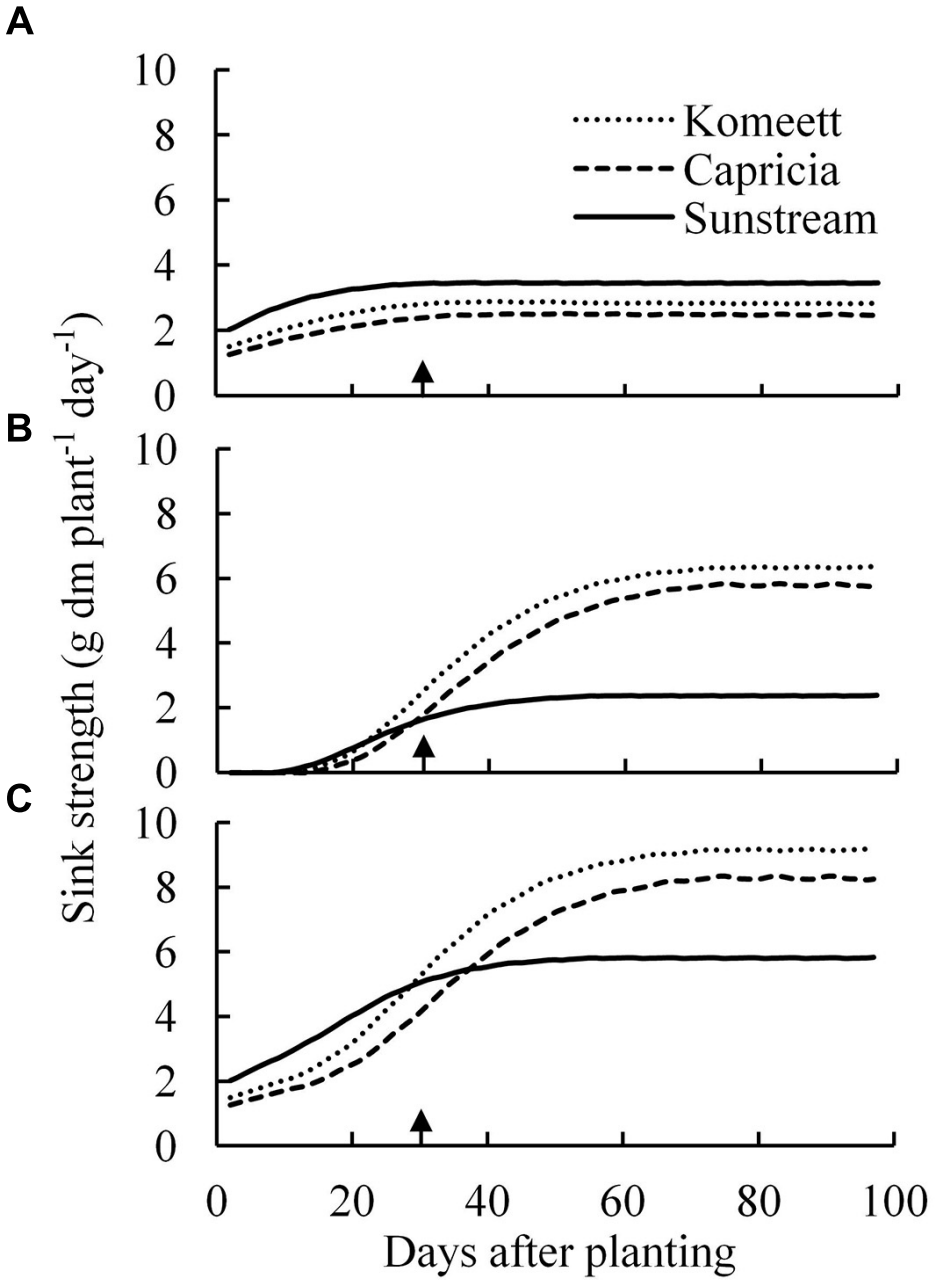
**Estimated vegetative **(A)**, total fruit **(B)**, and total plant **(C)** sink strength over time for the three tomato cultivars with standard fruit load**. Lines are moving averages over 5 days. Vegetative sink strength is the sum of the sink strengths of all the vegetative units of a plant; total fruit sink strength is the sum of the sink strengths of all fruits which are present on the plant; total plant sink strength is the sum of vegetative and total fruit sink strength. Arrow in X-axis indicates 30 days after planting. Fruit set started between 20–30 days after planting for the three cultivars. Therefore, the left side of arrow was defined as early growth stage, the right side of arrow was defined as fully fruiting stage.

The vegetative sink strength differed between cultivars and was highest for ‘Sunstream’ and lowest for ‘Capricia’ (**Figure 6A**). The total fruit sink strength was highest for ‘Komeet’ and lowest for ‘Sunstream’ (**Figure 6B**). Furthermore, the total fruit sink strength was initially low and soon increased to a plateau and kept constant onward. ‘Sunstream’ had highest total plant sink strength before 25 days after planting; thereafter, ‘Komeet’ had highest and ‘Sunstream’ had lowest total plant sink strength (**Figure 6C**).

**FIGURE 7:**
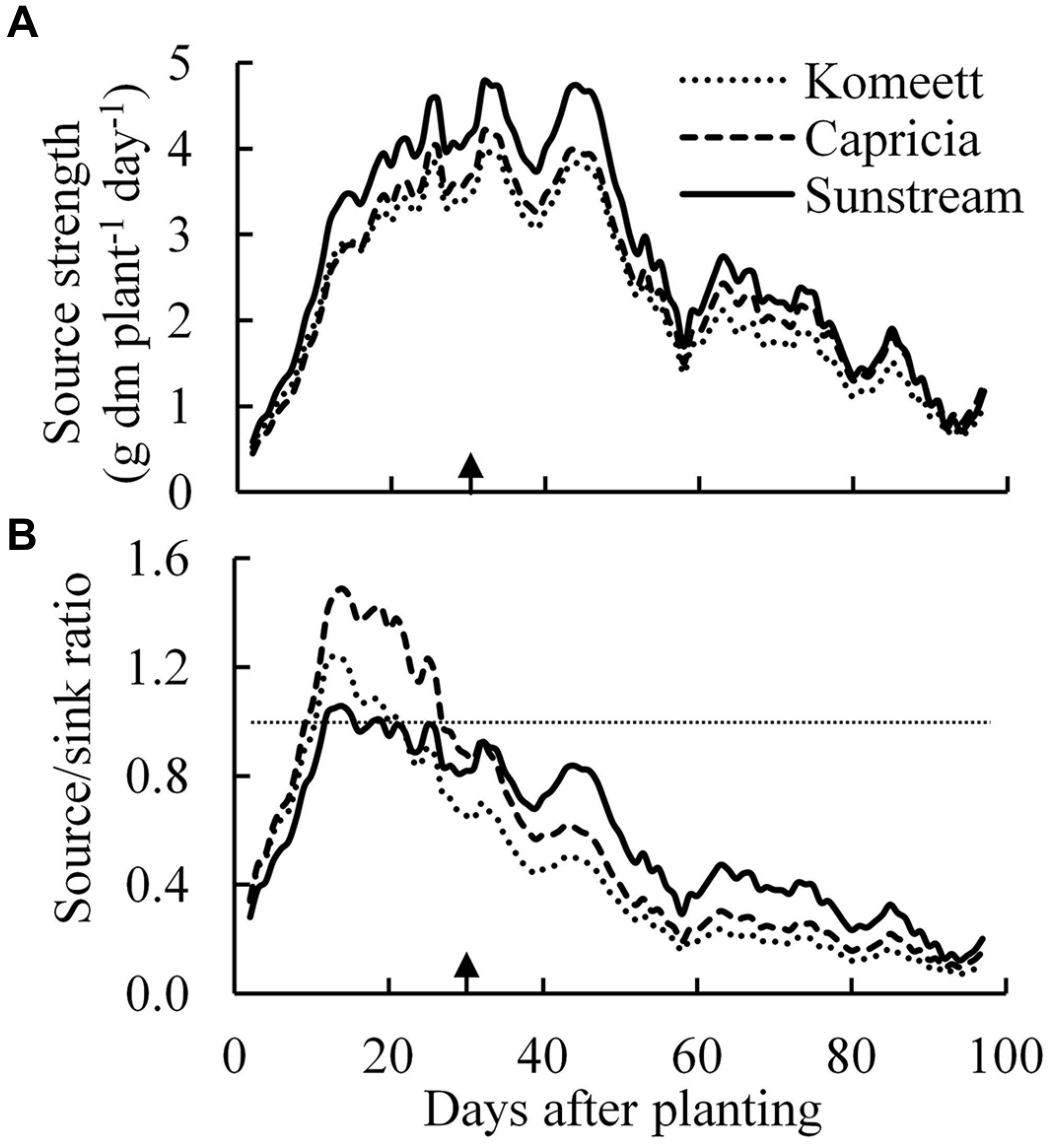
**Estimated source strength (crop growth rate; **A**) and source/sink ratio **(B)** over time for the three tomato cultivars with standard fruit load**. Lines are moving averages over 5 days. Dashed horizontal line in **(B)** represents a source/sink ratio of 1. Arrow in X-axis indicates 30 days after planting. Fruit set started between 20–30 days after planting for the three cultivars. Therefore, the left side of arrow was defined as early growth stage, the right side of arrow was defined as fully fruiting stage.

Source strength (crop growth rate) was initially low and increased drastically until about 30 days after planting (**Figure 7A**); it was decreasing from 45 days after planting onward until the end of the experiment. ‘Sunstream’ had higher source strength than the other two cultivars during a large part of the growing period (**Figure 7A**).

**FIGURE 8:**
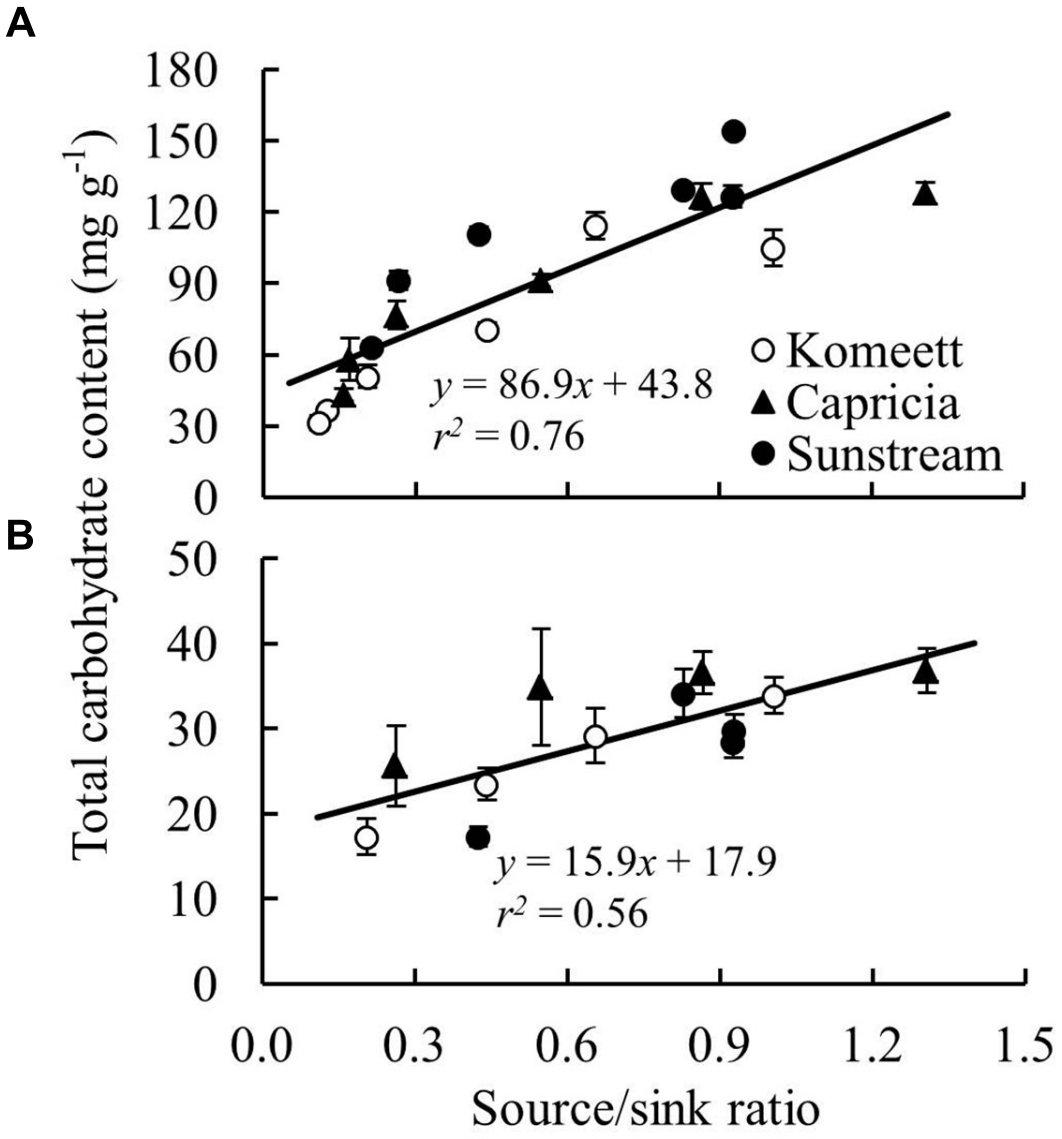
**The relationship between total carbohydrate content (sum of soluble sugar and starch content) and plant source/sink ratio in stems **(A)** and leaves **(B)** for three tomato cultivars with standard fruit load**. Lines represent linear regression line. In **(B)**, carbohydrate content determined at 81 and 97 days after planting (**Figure 4D**) were not included as these data were unexpected and remain unexplained.

Plant source/sink ratio was initially low (below 1) for all three cultivars, and it soon exceeded 1 in ‘Komeet’ and ‘Capricia,’ and came close to 1 in ‘Sunstream’ (**Figure 7B**). ‘Komeet’ had shorter duration of sink limitation than ‘Capricia,’ the source/sink ratio in ‘Komeet’ was also lower than in ‘Capricia.’ During the fully fruiting stage, source/sink ratio was lower than 1 for all three cultivars, ‘Sunstream’ had the highest and ‘Komeet’ had lowest source/sink ratio during this stage. Total carbohydrate content in stems and leaves over the three cultivars increased linearly with the source/sink ratio (**Figure 8**).

## Discussion

### Tomato Plants are Sink-Limited During their Early Growth Stage in Greenhouses Under High Irradiance

Young plants are likely to be sink-limited (Arp and Drake, 1991). Indeed, we found in our study that three types of tomato cultivars experienced a period of sink limitation or came close to sink limitation during their early growth stage (**Figure 7B**). Sink limitation during the early growth stage was caused by the low total plant sink strength (**Figure 6C**) combined with a fast increase in source strength (**Figure 7A**). This increase in source strength resulted from a fast increase in LAI. In addition, irradiance might also have played an important role, because sink limitation was observed during a period (early September) that plants received high natural irradiance to maintain a high rate of net photosynthesis compared to late autumn and winter months (**Figure 1**). The combination of the high irradiance and fast increase in LAI with limited reproductive organs during the early growth stage, resulted in plants not being able to use the extra assimilates, so that the high sugar content in stems was observed during this stage (**Figure 4A**). Tomato stems have been reported as an important storage organ for assimilates (Hocking and Steer, 1994), this is in line with our study that carbohydrate content in stems was higher than in leaves. Starch is predominantly utilized for diurnal carbon storage in leaves, it degrades to soluble sugar at night for mobilization and utilization (Smith and Stitt, 2007; Osorio et al., 2014), so that in stems sugar content was significantly higher than starch content (**Figure 4A**). In leaves the highest starch content was observed at 18 days after planting which was during the period of sink limitation (**Figure 4B**). Similarly, Plaut et al. (1987) and Nakano et al. (2000) also reported starch accumulation in leaves when sink limitation occurs.

Photosynthetic capacity often correlates with the source–sink balance (Iglesias et al., 2002; McCormick et al., 2006). In this study, net photosynthesis rates at 20 days after planting tended to be lower than at 28 days after planting when measured at the same conditions, although this was only significant for ‘Capricia’ (**Figure 5**). Sink limitation around 20 days after planting in combination with the high starch content in leaves (**Figure 4D**) might have led to a slight down-regulation of net photosynthesis (Nakano et al., 2000; Paul and Foyer, 2001; Iglesias et al., 2002). Irradiance induced acclimation could not play a role because the daily light sum was similar during this period (**Figure 1**). When young tomato plants not yet producing fruits were grown under elevated CO_2_, this resulted in photosynthetic acclimation (Yelle et al., 1989; Besford, 1993), which was probably caused by an imbalance in the supply and demand of assimilates. These studies further indicate that tomato plants are likely sink-limited during the early growth stage.

Source–sink balance is cultivar specific (**Figure 7B**). During the early growth stage cultivar differences in source/sink ratio were mainly due to differences in vegetative sink strength, as reproductive organs had hardly been formed or were still small and source strength was similar for the different cultivars (**Figure 7A**). ‘Sunstream’ had the highest vegetative sink strength (**Figure 6A**), and hence the lowest source/sink ratio during this period (**Figure 7B**). Wubs et al. (2009) also reported that cultivars with the smallest potential fruit size had the highest vegetative sink strength in sweet pepper. ‘Capricia’ had the lowest vegetative sink strength and consequently the highest source/sink ratio during the early growth stage (**Figure 7**).

### Fruiting Tomato Plants are Source-Limited and Source/Sink Ratio Negatively Correlates with the Potential Fruit Size When Standard Fruit Load is Maintained

A major change in plant development is the switch from vegetative growth to generative growth. This change was also followed by a marked change in source–sink balance in the current experiment (**Figure 7B**). For all three cultivars, source/sink ratio was below 1 during the fully fruiting stage (**Figure 7B**), suggesting source limitation. This is also supported by the observation that half fruit load treatment did not influence the total plant dry weight (**Table 1**). This result is in agreement with many previous studies that fruiting tomato plants grown in greenhouses are source-limited (De Koning, 1994; Cockshull and Ho, 1995; Heuvelink and Buiskool, 1995; Matsuda et al., 2011; Qian et al., 2012). Our results contradicts those of Dueck et al. (2010) who estimated that cherry tomato is most likely sink-limited. The source/sink ratio of fruiting tomato plants in this study (average source/sink ratio was 0.17–0.33 from 50 days after planting onward for all three cultivars) was lower than the value (about 0.5) which has been reported by De Koning (1994) and Heuvelink (1996b). This is mainly attributed to the low irradiance level during the fully fruiting stage (**Figure 1**). Furthermore, De Koning (1994) reported that tomato potential fruit growth rate positively correlates with the irradiance level. In this study, the potential fruit growth rate used for sink strength estimation was mainly determined from those fruits that developed under relatively high irradiance level (in September and early October). This might have slightly overestimated the sink strength of the plants during the low irradiance period. Additionally, fruit position within a truss also plays a role, i.e., potential growth rate of the first six fruits was higher than the other fruits within a truss (De Koning, 1994). In this study, the potential growth rate of a single fruit was estimated from the first three fruits within a truss, therefore, the sink strength of ‘Sunstream’ (10 fruits per truss) might have been overestimated. Although there were several pitfalls for the estimation of sink strength in this study, the average fresh weight of harvest-ripe fruits from the plants with half fruit load was 1.4, 2.2, and 2.3 times higher than the fruits from plants with standard fruit load in ‘Sunstream,’ ‘Capricia,’ and ‘Komeet,’ respectively. This clearly indicates that fruiting tomato plants were source-limited for all three cultivars.

During the fully fruiting stage, total fruit sink strength played a pivotal role in determining the source/sink ratio, because differences in source strength and vegetative sink strength between cultivars were small (**Figure 6**). ‘Sunstream’ (cherry tomato) showed the lowest total fruit sink strength, while ‘Komeet’ (large-sized fruits) showed the highest total fruit sink strength (**Figure 6B**). Hence, a negative correlation between potential fruit size and source/sink ratio during the fully fruiting stage was observed when standard fruit load was maintained (**Figure 7B**).

Plant carbohydrate content is positively correlated with the source–sink balance (Schnyder, 1993; Iglesias et al., 2002; Li et al., 2002). In line with these results a linear relationship between plant source/sink ratio and total carbohydrate content in stems (**Figure 8A**) as well as in leaves (**Figure 8B**) was observed, which relationship was independent of cultivar. Carbohydrate content (i.e., sugar content in stems and starch content in leaves) during the fully fruiting stage was generally lower than during the early growth stage (**Figure 4**). Among the three cultivars, ‘Sunstream’ showed the highest source/sink ratio and consequently the highest sugar content in stems during the fully fruiting stage, while ‘Komeet’ showed the lowest source/sink ratio and sugar content in stems (**Figure 4A**). The positive correlation between carbohydrate content in stems and source/sink ratio was also observed by Hall and Milthorpe (1978) and Ho et al. (1983). In leaves, the sudden increase in starch content at 80 days after planting in ‘Komeet’ and to a lesser extent at 97 days after planting in the other two cultivars was unexpected as source/sink ratio was very low during this period (**Figure 7B**); this remains unexplained.

## Implications

Fruiting tomato plants were strongly source-limited even for cherry tomato (‘Sunstream’) as indicated by the low source/sink ratio (average source/sink ratio from 50 days after planting onward was 0.17–0.33 for three tomato cultivars). Despite the application of supplementary lighting (162 μmol m^-2^ s^-1^ PAR; maximum 10 h per day), irradiance in the greenhouse declined due to decreased natural irradiance toward the winter. Therefore, extending the duration or increasing the PAR intensity of supplementary lighting in combination with maintaining lower fruit load could be considered to better balance source and sink strength. Early growth stage tomato plants showed sink limitation as indicated by a source/sink ratio exceeding 1. For sink-limited plants, giving more light will not increase plant growth as surplus assimilates in leaves could down-regulate leaf photosynthesis.

## Conclusion

Our conclusions are: (1) tomato plants are sink-limited during the early growth stage under high irradiance; (2) under commercial crop management fully fruiting tomato plants are source-limited, this is even the case for small fruited cherry tomato; (3) during the fully fruiting stage of tomato cultivars, the source/sink ratio is negatively correlated with the potential fruit size when standard fruit load is maintained; and (4) carbohydrate content in tomato stems and leaves increases linearly with the plant source/sink ratio.

## Author Contributions

TL carried out the measurements, data analysis, and drafted the manuscript. LM and EH made substantial contributions to conception and experiment design, and critically revised the manuscript.

## Conflict of Interest Statement

The authors declare that the research was conducted in the absence of any commercial or financial relationships that could be construed as a potential conflict of interest.

## Abbreviations

LAI: leaf area index
PAR: photosynthetic active radiation
SLA: specific leaf area.
